# Ginsenosides in the management of depression: a comprehensive pharmacological review

**DOI:** 10.1186/s13020-026-01416-x

**Published:** 2026-05-15

**Authors:** Min Ren, Xin Tao, Hong Chang, Hui-Chang Bi, Simon Ming-Yuen Lee, Jian-Bo Wan

**Affiliations:** 1 The State Key Laboratory of Mechanism and Quality of Chinese Medicine, Institute of Chinese Medical Sciences, University of Macau, Room 6034, Building N22, Avenida da Universidade, Taipa, Macao, 999078 China; 2 NMPA Key Laboratory for Research and Evaluation of Drug Metabolism & Guangdong Provincial Key Laboratory of New Drug Screening & Guangdong-Hongkong-Macao Joint Laboratory for New Drug Screening, School of Pharmaceutical Sciences, Southern Medical University, Guangzhou, China; 3 Department of Food Science and Nutrient, The Hong Kong Polytechnic University, Hung Hom, Kowloon, Hong Kong, SAR China; 4 Guangdong-Hong Kong-Macao Joint Laboratory for New Drug Screening, University of Macau, Macao, SAR China

## Abstract

Depression is a prevalent and debilitating psychiatric disorder that is frequently accompanied by chronic conditions such as cancer, cardiovascular diseases, and neurological disorders. Despite the availability of various pharmacological treatments, their limited efficacy and frequent side effects have prompted growing interest in natural compounds with antidepressant potential. *Panax ginseng*, a traditional herbal medicine widely used in East Asia, contains diverse bioactive components, among which ginsenosides are recognized as the principal active constituents. Ginsenosides, primarily classified into dammarane-type and oleanane-type saponins, exhibit antidepressant-like effects through multiple interconnected biological mechanisms. These include modulation of monoaminergic neurotransmission, regulation of the hypothalamic–pituitary–adrenal (HPA) axis, promotion of neurogenesis and synaptic plasticity, mitigation of neuroinflammation and oxidative stress, and restoration of gut microbiota homeostasis. Recent investigations also highlight enhanced bioavailability and therapeutic promises of rare ginsenosides, such as ginsenosides Rg3, Rk1 and Rg5, with fewer sugar moieties, suggesting unique advantages for clinical application. This review consolidates current evidence on the pharmacological activities, molecular targets, and therapeutic potential of ginsenosides in the management of depression. By integrating findings from experimental and limited clinical studies, it aims to provide a rational scientific framework to inform future investigation and development of ginsenoside-based strategies for depression, while emphasizing the need for further rigorous clinical validation.

## Introduction

Depression is a prevalent and disabling psychiatric disorder that often co-occurs with chronic medical conditions such as cancer, cardiovascular disease, metabolic disorders, and neurological disorders [1]. According to the World Health Organization (WHO), depression is characterized by persistent low mood or loss of interest or pleasure lasting at least two weeks, typically accompanied by disturbances in sleep, appetite, weight, and energy levels [2]. Globally, depression affects an estimated 3.8% of the population and remains a leading cause of disability, with suicide representing one of its most devastating outcomes [3]. The coronavirus disease 2019 (COVID-19) pandemic has further magnified this burden, as the virus’s impact on the central nervous system (CNS) has been linked to mood disturbances, including depression and anxiety [4]. Although several pharmacological therapies are available, fewer than half of patients achieve adequate symptom remission, and many experience relapses or adverse effects. Common limitations of current therapies, such as delayed onset of action, reduced long-term efficacy, and safety concerns [5], have spurred growing interest in naturally derived compounds as promising, safer alternatives for managing depressive disorders.

**Fig. 1:**
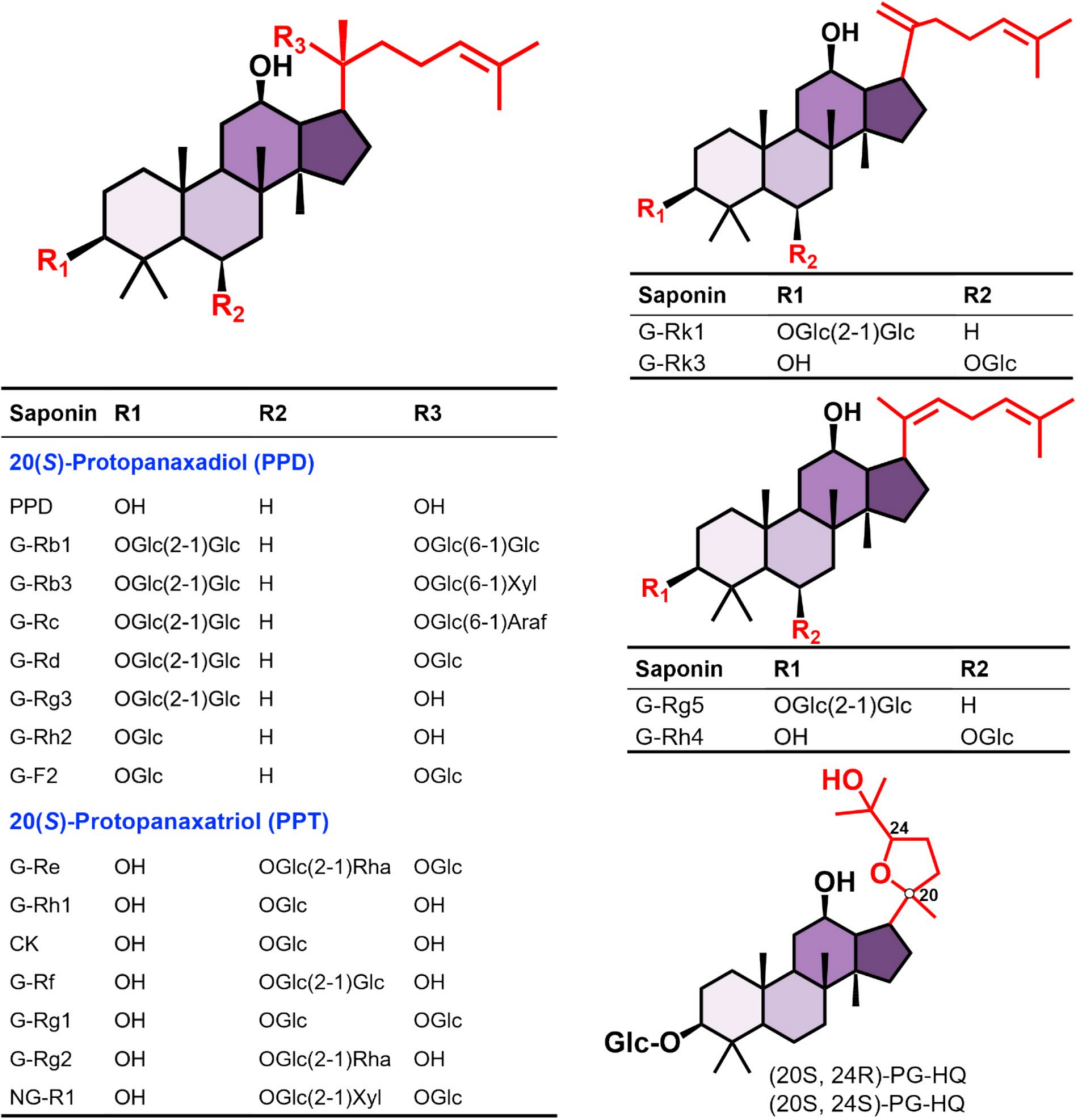
Representative chemical structures of major ginsenosides reported to possess antidepressant properties. G, ginsenoside; CK, compound K; NG, notoginsenoside; PG, pseudo-ginsenoside

*Panax ginseng* (Ginseng), a medicinal herb of the Araliaceae family, has been used for centuries in traditional Eastern medicine as both a restorative tonic and a therapeutic agent for various disorders [6]. It contains a wide spectrum of bioactive compounds, including ginsenosides, polysaccharides, proteins, sterols, and essential oils [7, 8]. Among these constituents, ginsenosides are recognized as the principal active components responsible for the majority of ginseng’s pharmacological actions [9–11]. Structurally, ginsenosides are glycosylated triterpenoid saponins composed of a 17-carbon steroid-like backbone with attached sugar moieties [12]. Ginsenosides are primarily classified into two chemical types: dammarane-type, which includes protopanaxadiol (PPD) and protopanaxatriol (PPT) subgroups, and oleanane-type, which contains oleanolic acid (ginsenoside Ro) and ocotillol (pseudoginsenoside F11) derivatives (Fig. 1) [13, 14]. They can also be categorized based on their natural abundance as macro or rare ginsenosides**.** Rare ginsenosides, which typically represent less than 0.1% of total ginsenoside content, have attracted increasing attention for their superior pharmacological activity [11, 15]. Owing to their enhanced bioavailability and biological potency, these rare ginsenosides contribute significantly to the antidepressant-like effects of ginseng.

**Fig. 2:**
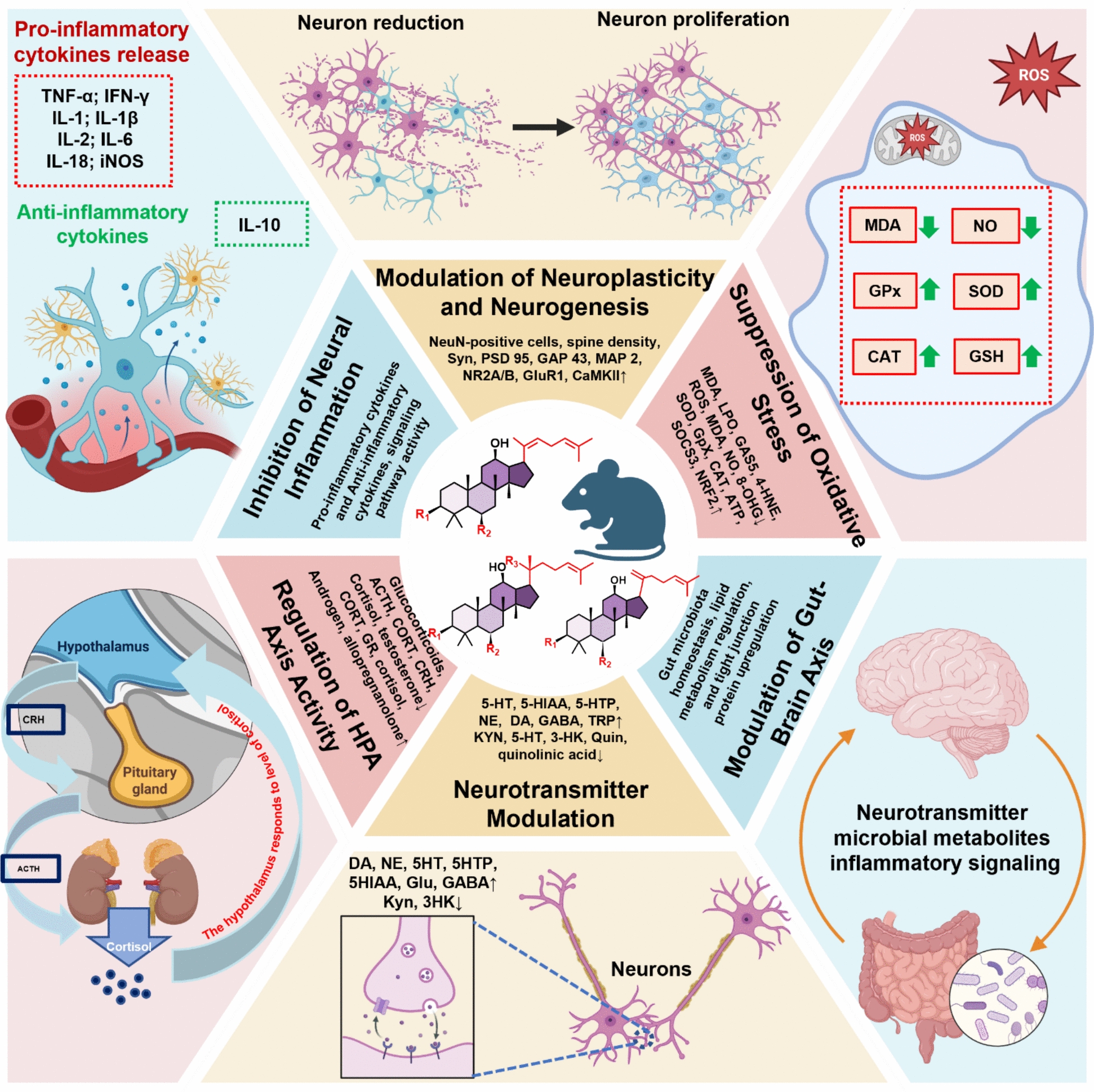
Representative molecular targets involved in the pathogenesis of depression and modulated by ginsenosides

A growing body of preclinical studies has provided substantial evidence supporting the antidepressant-like effects of ginsenosides. In animal models, these compounds consistently reduce immobility time in the forced swim and tail suspension tests, while increasing sucrose preference and locomotor activity, which are the behavioral indicators of amelioration of stress‑related. Mechanistic studies suggest that ginsenosides act through multiple, interrelated biological pathways. They modulate monoaminergic neurotransmission, normalize hypothalamic–pituitary–adrenal (HPA) axis function, promote neurogenesis and synaptic plasticity, suppress neuroinflammation, mitigate oxidative stress, and influence Gut–Brain Axis interaction (Fig. 2). Despite considerable progress, the precise molecular mechanisms underlying these effects remain incompletely understood. Accordingly, this review comprehensively summarized the current available evidence on their pharmacological activities and underlying antidepressant mechanisms, providing a theoretical and empirical framework for the development of ginsenoside-based natural antidepressants.

## Search strategy and inclusion criteria

Relevant literatures published between 2013 and 2025 were retrieved from PubMed and Web of Science using the following search terms in title field: (“Ginsenosides” OR “*Panax*” OR “Ginseng” OR “Ginseng saponin”) AND (“Depression” OR “Depressive disorder” OR “Depress” OR “Emotional disorder” OR “Psychological disorder” OR “Psychological distress” OR “Emotional distress” OR “Emotional stress”). Additional relevant studies were included through manual searches of reference lists from selected articles. Studies were considered eligible if they met the following criteria: (*i*) the intervention involved ginsenosides or traditional Chinese medicine formulations containing ginsenosides (e.g., *Kai-xin-san*); (*ii*) The research included in vivo or in vitro studies; (*iii*) the study investigated the mechanisms underlying the antidepressant-like effects of ginsenosides; (*iv*) the articles were published in English; and (*v*) relevant references not captured by the initial search were also included following manual review.

## Ginsenosides in the alleviation of depression-like behaviors

Over recent decades, ginsenosides have attracted growing attention for their neuroprotective and psychopharmacological properties, particularly for their potential to alleviate depressive symptoms with minimal toxicity. In this review, evidence from 74 studies involving 20 distinct ginsenosides and 9 ginseng-related herbal preparations demonstrating antidepressant-like activity is summarized (Table 1). The chemical structures of ginsenosides involving anti-depressant activities are shown in Fig. 1.

**Table 1:** Representative ginsenosides and ginsenoside-containing herbal formulas investigated for their potential antidepressant-like effects.

Ginsenoside	Animal model	Dosage (Route of administration)	Efficacy	Mechanisms	Refs.
20(*S*)-Protopanaxadiol	CUMS-exposed male C57BL/6 J mice	20 and 40 mg/kg (*p.o.*) for 3 weeks	SPT↑; BW↑; immobility time in FST & TST & OFT↓; residence time in target quadrant of Morris water maze↑	Neurotransmitter modulation (5-HT↑ in serum); Suppression of oxidative stress (MDA↓ in serum; adenosine triphosphate (ATP) ↑ in hippocampal); Enhancement of neuroplasticity and neurogenesis (DRP1↓& SIRT1, PGC-1α, BDNF↑ in hippocampus)	[59]
Rb1	CUMS-exposed ICR mice	0.9 mM in 0.5% carboxymethyl cellulose sodium (CMC-Na, *i.g.*)	SPT↑; immobility time in FST & TST↓	Modulate Neuroplasticity and Neurogenesis (miR-134 ↓& NeuN-positive cells, spine density, Syn, PSD-95, GAP-43, MAP-2, NR2A/B, GluR1, CaMKII↑); Activation of BDNF–TrkB–AKT–ERK–GSK3β–CREB signaling in the hippocampus	[53]
Rb1 + Berberine	Streptozotocin-injected, CUMS-exposed SD rats	150 mg/kg berberine + 20 mg/kg Rb1 (*i.g.*) for 4 weeks	SPT↑; immobility time in FST & TST↓; EPM open-arm time↑; OFT locomotion↑	Regulation of HPA axis (Cortisol, ACTH↓); Modulate Neuroplasticity and Neurogenesis (BDNF↑ in hippocampus)	[16]
Rb1	LPS (1 mg/kg, *i.p.*)-induced acute depression-like behavior in ICR mice	10 and 20 mg/kg (*p.o.*) for 11 days	Immobility time in FST & TST ↓	Inhibition of Neural Inflammation (TNF-α, IL-1β, IL-6↓ in serum; p-p65/p65, p-p38 MAPK/p38 MAPK↓ & GR↑ in hippocampal); Neurotransmitter modulation (KYN, KYN/TRP ratio, 5-HT↓ & TRP, 5-HT1A receptor↑ in hippocampus)	[48]
Rb1	CSDS-exposed CD1 and C57BL/6 J mice	35 and 70 mg/kg (*p.o.*) for 33 or 32 days	SPT↑; Immobility time in FST & TST↓; Duration in interaction zone↑; Social interaction radio↑	Modulate Neuroplasticity and Neurogenesis (DCX⁺ cell number, DCX mRNA, p‑AKT/AKT, p‑CREB/CREB, p‑ERK/ERK ratio, BDNF protein↑ in hippocampus); Suppression of oxidative stress (LPO↓& SOD, CAT↑ in hippocampus); Inhibition of neural inflammation (Iba-1⁺ cell number, IL-1β, NLRP3, ASC, cleaved-Caspase-1, TNF-α, IL-18↓& IL-1β, SIRT1 immunoreactivity, SIRT1 protein, Nrf2, HO-1 protein expression↑ in hippocampus)	[18] [21]
Rb1	CUMS-exposed SD rats	10 mg/kg (*i.g.*) for 6 weeks	SPT↑, Immobility time in FST↓; Duration in interaction zone↑	Inhibition of neural inflammation (glial fibrillary acidic protein (GFAP)/Caspase-1, PINK1, p62, NLRP3, ASC, pro-Caspase-1, Caspase-1, IL-1β, pro-IL-1β↓& Protein TH, LC3 II/LC3 I↑ in hippocampal); Modulate Neuroplasticity and Neurogenesis (NeuN/Caspase-3↓ & Number of synapses, dendritic spines, total dendritic length, Syn, PSD-95, GAP-43↑)	[68]
Rb1	CRS-exposed ICR mice	10 mg/kg (*i.p.*) for 2 weeks	Immobility time in FST & TST↓; Duration in interaction zone↑	Inhibition of neural inflammation (TNF-α, Iba-1↓& IL-1β↑ in hippocampus); Modulate Neuroplasticity and Neurogenesis (BDNF, p-AKT/AKT↑ in hippocampus)	[26]
Rb1 Rb1 + fluoxetine Rb1 + caffeine	CUMS-exposed Wistar rats; 5-HTP (200 mg/kg) + reserpine (4 mg/kg)-induced ICR mice	4, 8, 10 mg/kg (*i.p.*) for 1 week, 1 mg/kg fluoxetine (*i.p.*) + 4 mg/kg Rb1 (*p.o.*), 5 mg/kg caffeine (*i.p.*) + 4 mg/kg Rb1 (*p.o.*)	Immobility time in FST & TST↓; Duration in interaction zone↑; number of head twitches↓; Score of ptosis↓; hypothermia and locomotor activity↑	Neurotransmitter modulation (5-HT, 5-HIAA, NE, DA↑ in hippocampal)	[17]
Rb1	CMS-exposed C57BL/6 J mice	20 mg/kg (*i.g.*) for 4 weeks	Immobility time in FST & TST↓; weight gain↑; SPT↑	Inhibition of neural inflammation (TNF-α, IL-1β, Density and area of Iba1 + cells↓& the number and total length of processes per Iba1 + cell, TGF-β, Arg-1↑in hippocampus and cortex, DG; granular cell layer (GCL) volume, GCL thickness, PPARγ, p-PPARγ↑ in hippocampus)	[31]
Rb1	CRS-exposed C57BL/6 J mice	35 mg/kg (*i.g.*) for 4 weeks	Immobility time in FST↓	Inhibition of neural inflammation (Iba1 + cells, TLR4, pNF-κB,C3↓ in hippocampus); Modulate Neuroplasticity and Neurogenesis (Density of dendritic spines, Syn, PSD-95↑ in hippocampus);	[27]
Rb1 Rb1 + fluoxetine Rb1 + reboxetine Rb1 + bupropion Rb1 + Mk-801 Rb1 + baclofen	ICR mice	5, 10, 20 mg/kg (*p.o.*) Rb1, 1 mg/kg fluoxetine (*i.p.*) + 5 mg/kg Rb1 (*p.o.*), 2.5 mg/kg reboxetine (*i.p.*) + 5 mg/kg Rb1 (*p.o.*), 10 mg/kg bupropion (*i.p.*) + 5 mg/kg Rb1 (*p.o.*), 0.05 mg/kg Mk-801 (*i.p.*) + 5 mg/kg Rb1 (*p.o.*), 0.1 mg/kg baclofen (*i.p.*) + 5 mg/kg Rb1 (*p.o.*),	Immobility time in FST↓	Neurotransmitter modulation (5-HT, 5-HIAA, NE, GABA, glutamates, DA↑ in hippocampal and cortex)	[83]
Rb3, Rg3, Rh2, compound K, and 20(S)-protopanaxadiol	Despair model of NIH mice	50 and 100 mg/kg (*p.o.*) Rb3 for 1 h, 7 or 14 days	Immobility time in FST & TST↓	Neurotransmitter modulation (NE↑ in hippocampal and cortex); Regulation of HPA axis activity (ACTH, CORT↓ in serum)	[82]
Rc	L-α-aminoadipic acid (L-AAA, 100 μg/mL)-induced C57BI/6 mice	20 mg/kg (*p.o.*) for 7 days	Immobility time in FST & TST↓	Inhibition of neural inflammation (Number and area of Iba1^+^ cells, IBA-1, TNF α, IL-6, Lipocalin-2, caspase-3↓ & TGF-β, GFAP^+^ cells, GFAP, Bcl-2↑ in cortex, DG; GCL volume, GCL thickness, PPARγ, p-PPARγ↑ in hippocampus)	[44]
Rd	CUMS-exposed SD rats; Despair model of ICR mice	10 and 20 mg/kg (*i.g.*) for 14 days	SPT↑; Immobility time in FST & TST↓; Distance, frequency, time and velocity in interaction zone↑;	Modulate Neuroplasticity and Neurogenesis (Syn, PSD95, PI3K, AKT, mTOR mRNA, HIF-α, VEGF, VEGFR2↑ in hippocampal)	[69]
Rd, fRG, RG, Protopanaxatriol	Immobilization stress (IS) or EC-exposed C57BL/6 mice	10 and 25 mg/kg (*i.g.*) RG for 5 days, 10 and 25 mg/kg (*i.g.*) fRG for 5 days, 5 mg/kg (*i.g.*) protopanaxatriol for 5 days	Duration and enteries in open arms↑; Duration in light↑; Immobility time in FST & TST↓	Modulate Neuroplasticity and Neurogenesis (p-p65/p65, NF-κB^+^/Iba1^+^, NF-κB^+^/CD11c^+^↓ & p-CREB/CREB, BDNF, BDNF^+^/NeuN^+^ cells↑ in hippocampal); Inhibition of Neural Inflammation (CORT, IL-6↓ in blood; Length, myeloperoxidase (MPO) activity, TNF-α, IL-6 and NF-κB↓ in colon); Modulation of Gut-Brain Axis (Gut dysbiosis↓, gut microbial abundances change)	[75]
Rd	LPS (0.83 mg/kg, *i.p.*)-induced ICR mice	4, 12 and 40 mg/kg (*i.p.*) in 0.3% CMC for 2 weeks	SPT↑; Immobility time in FST & TST↓	Modulate Neuroplasticity and Neurogenesis (Nissl's body ratio, Damaged neuron ratio↓&BrdU^+^, PSD-95, SYP, Dendritic spine density↑ in hippocampal); Inhibition of Neural Inflammation (Iba1, JMJD3, TLR4, MyD88, p-PI3K/PI3K, p-AKT/AKT, p-p65/p65↓ in hippocampal)	[54]
Re	CUMS-exposed C57BL/6 J mice	20 and 40 mg/kg (*i.g.*) for 2 weeks	SPT↑; Immobility time in TST↓; Distance, time and number of rearing in interaction zone↑;	Inhibition of Neural Inflammation (p62, NLRP3, ASC, Caspase-1↓& PINK1, Parkin, LC3, Beclin1↑ in cortex)	[55]
Re	Reserpine-exposed ICR mice	10, 30 and 90 mg/kg (*i.p.*) for 1 week	Immobility time in FST & TST↓	Suppression of Oxidative Stress (MDA↓& SOD↑ in hippocampal); Inhibition of Neural Inflammation (IL-1β, IL-6 and TNF-α↓ & Cleaved Caspase 3/Caspase 3, PINK1, Parkin, LC3, Beclin1↑ in hippocampal); Modulate Neuroplasticity and neurogenesis (p-ERK/ERK, p-CREB/CREB, p-TrkB/TrkB, p-AKT/AKT, BDNF↑ in hippocampal)	[43]
Rf	Chronic constriction injury (CCI)-exposed SD rats	0.5, 1.5 and 3 mg/kg (twice/day, *i.p.*) for single injection or 3 weeks	Immobility time in FST↓; paw withdrawal threshold↑	Inhibition of Neural Inflammation (IL-1β, IL-6, IL-18, IL-1RA↓ in spinal cord; IL-1β, IL-6, IL-18BP↓& IL-1RA, IL-10↑ in dorsal root ganglion);	[154]
Rf	L-AAA (1.2 μg/mouse)-induced C57BI/6 mice	20 mg/kg (*p.o.*) for 1 week	Immobility time in FST & TST↓	Modulate Neuroplasticity and neurogenesis (GFAP, Ki-67↑ in hippocampal and cortex)	[45]
Rg1 + Run	LPS-induced Wistar rats	40 mg/kg (*i.p.*) for 2 weeks	Immobility time in FST↓; SPT↑; Duration in interaction zone↑ Duration and enteries in open arms↑	Inhibition of Neural Inflammation (glial cells, IL-1β, IL-6, interferon gamma (IFN-γ) mRNA↓ in hippocampal and cortex); Modulate Neuroplasticity and neurogenesis (Density of mushroom spines, Number of synapses and synaptic vesicles, p-CREB, BDNF, SYT1, PSD-95↑ in hippocampal; p-CREB, BDNF, PSD-95↑ in cortex)	[46]
Rg1	CMS-exposed C57BL/6 J mice	20 mg/kg (*i.p.*) for 3 weeks	SPT↑; BW↑; immobility time in FST & TST↓; Time & Frequency in center↑	Modulate Neuroplasticity and neurogenesis (microglia cells, Cell solidity, ASC^+^ microglia↓& branches cells, Iba1^+^ cells, Cell perimeter, Critical radius, the dendritic maximum of microglia, BrdU ^+^, DCX ^+^ cells, BrdU^+^-DCX^+^ cells/BrdU^+^ cells, nascent neurons and branch lengths, mature neurons↑ in hippocampus); Inhibition of Neural Inflammation (Numbers of Iba1^+^ cells, area of Iba1^+^ cells, ASC^+^/Iba1^+^ cells, ASC↓& Numbers of branches, Critical radius of microglia, dendritic maximum of microglia, BrdU^+^ cells, BrdU^+^-DCX^+^ cells, NeuN^+^ cells, number of nascent neurons, nascent neurons branch length↑ in hippocampus)	[32]
Rg1	CRS-exposed SD rats	20 mg/kg (*i.g.*) for 4 weeks	SPT↑; Immobility time in FST↓; Locomotor activities↑	Suppression of Oxidative Stress (GAS5↓ & ATP, copy number of mitochondrial DNA, mitochondrial length/wide ration, SOCS3, NRF2, EZH2↑ in hippocampal); Inhibition of neural inflammation (TNF-α, IL-6, IL-1β↓ in hippocampus)	[28]
Rg1	CUS-exposed Wistar rats	40 mg/kg for 4 weeks (*i.p.*) or 8 weeks (*i.g.*)	SPT↑; BW↑; immobility time in FST↓; Distance and number of rearing in interaction zone↑; Locomotor activities↑	Modulate Neuroplasticity and neurogenesis (uptake of radioactivity↓ & YAP, p-YAP, p-YAP/YAP, Cx43, p-Cx43, p-Cx43/Cx43, GFAP, Cytoskeleton size of astrocytes↑ in the brain; Lucifer Yellow spreading area↑); Inhibition of Neural Inflammation ((IL-1β, TNF-α, caspase-1, IL-2, IL-6, IL-18, CORT↓ in serum; Ubiquitinated Cx43, caspase-1, IL-1β, IL-2↓ in cortex)	[36] [37]
Rg1	LPS (2 mg/kg, *i.p.*), single-prolonged stress (SPS), CS/US-CS-exposed C57BL/6 J mice	10, 20 and 40 mg/kg (*i.p.*) for 2 weeks	Immobility time in FST & TST↓; Frezing time↓	Inhibition of Neural Inflammation (PSD95, Arc, GluA1, GFAP, Iba-1^+^, TNF-α and IL-1β, GluN2A, Kir4.1, GFAP↓ in hippocampus)	[47]
Rg1	CUS-exposed SD rats	20 mg/kg (*i.p.*) for 45 days or 20 mg/kg (twice/day, *i.g.*) for 3 days	SPT↑; Immobility time in FST↓; Latency of feed↓	Modulate Neuroplasticity and Neurogenesis (Cx 43, Cell viability↑ & gap width between astrocytes↓ in hippocampus; Diffusion distance of Lucifer yellow↑);	[38] [39]
Rg1	CSDS-exposed CD1 and C57BL/6 J mice	20 and 40 mg/kg (*p.o.*) for 34 days	SPT↑; Immobility time in FST & TST↓; Duration in interaction zone↑	Suppression of Oxidative Stress (SIRT1, phosphorylated ERK1/2, DCX ^+^ cells, DCX↑ & phosphorylated JNK, p38↓ in hippocampus); Inhibition of Neural Inflammation (Iba1 + cells, TNF-α, IL-6, IL-1β, p-p65, iNOS, cyclooxygenase-2 (COX-2), cleaved caspase-3, cleaved caspase-9, acetylated p65↓ in hippocampus; TNF-α, IL-6, and IL-1β↓ in serum)	[22]
Rg1	CUMS-exposed SD rats	20 and 40 mg/kg (*i.p.*) for 3 weeks	SPT↑; BW↑; immobility time in FST & TST↓; Grooming, crossing and rearing↑	Inhibition of Neural Inflammation (p-NF-κB, NLRP3, ASC-1, Caspase-1, TNF-α, IL-6, IL-1β↓ in cortex; CORT↓ in serum)	[70]
Rg1	CUMS-exposed Wistar rats	40 mg/kg (*i.p.*) for 5 weeks	SPT↑; Immobility time in FST & TST↓; Time in central↑	Suppression of Oxidative Stress (BCL2-associated X protein (Bax), caspase 3, caspase 9, superoxide production, oxidative base damage levels, mitochondrial superoxide levels, 4-HNE, ROS, MDA and NO↓& SOD, GpX, density of positive cleaved caspase 3, NeuN double-labeled cells↑ in hippocampus); Inhibition of Neural Inflammation (synapse, GFAP^+^ astroglia, activated microglia, TNF-α, IFN-γ, IL-1β, CD11b, CD45↓in hippocampus); Modulate Neuroplasticity and neurogenesis (BDNF, miR-134↓ & synapse density of individual neurons, Syn, BDNF, p-CREB/CREB, p-PKA/PKA, p-CREB↑ in basolateral amygdala region; miR-134↓ & spine density of individual neurons, synapse density of individual neurons, p-cofilin, Limk1, BDNF, p-CREB/CREB, p-ERK1/2/p-ERK1/2↑ in cortex)	[113] [155] [156] [157] [158]
Rg1	CUMS-exposed SD rats Imatinib-treated Kunming mice	5, 10, 20 and 40 mg/kg (*p.o.*) for 4 weeks	SPT↑; BW↑; Immobility time in FST & TST↓; Sleep duration↑; rearing and residence in central region time↑	Regulation of HPA axis activity (testosterone↓ & CORT↑ in serum; GR↑ in hippocampus; Androgen, AR + cells↑ in cortex)	[19]
Rg1	CUS-exposed SD rats	5, 10 and 20 mg/kg (*i.g.*) for 4 weeks	SPT↑; Immobility time in FST↓;	Inhibition of Neural Inflammation (gap width between astrocytes↓ & astrocytes coupling, distance of astrocyte gap junctions fluorescent dye LY, Cx43, Cx43/GFAP-immunoreactive puncta↑ in cortex)	[40]
Rg1	LPS or CSDS-exposed C57BL/6 J mice	20 mg/kg (*i.p.*) for 4 days or weeks	SPT↑; Immobility time in FST↓; Weight loss↓	Inhibition of Neural Inflammation (IL-6↓& TNF-α↑ in blood; Ly6Chi monocytes, migration of peripheral Ly6Chi monocytes, GFAP↓ in brain; CCL2, CCL3, IL-1β, CXCL10↓ in cortex; IL-1β↓ in hypothalamus; IL-1β↓ in cortex)	[23]
Rg1	CRS-exposed C57BL/6 J mice	5 and 10 mg/kg (*i.p.*) for 3 weeks	SPT↑; Immobility time in FST & TST↓; Total distance↑; time and enteries in open arm↑; Ambulatory time zone↑ Number of platform area crossings and Quadrant occupancy in the space and reversal exploring test stage↑; Mean escape latency↓	Modulate Neuroplasticity and neurogenesis (NR2A, PSD95, BDNF, Bcl-2↑ & Bax, p-MST1/MST1, p-LATS1/LATS1, p-YAP/YAP↓ in hippocampus)	[29]
Rg1	Kyn (2 mg/kg, *i.p.*) or CMS-exposed C57BL/6 J mice	20 and 40 mg/kg (*i.p.*) for 1 week	SPT↑; Immobility time in FST & TST↓	Neurotransmitter modulation (Kyn↓ in serum, hippocampus, liver; KAT2, KMO↑ in liver)	[33]
Rg1	UMS-exposed C57BL/6 J mice	20 and 40 mg/kg (*i.p.*) for 2 weeks	Immobility time in FST & TST↓; Marble-burying test↑; social interaction test (SIT)↑	Modulate Neuroplasticity and neurogenesis (DCX^+^ cells, CamkIIα, indole-3-acetic acid↑ in hippocampus; indole-3-acetic acid↑ in serum, hypothalamus)	[42]
Rg1	Postpartum depressed mice and gestation	150 mg/kg (*i.p.*) for 9 days	Immobility time in FST & TST↓; Crossing↑	Suppression of Oxidative Stress (MDA↓ & SOD, GPx, CAT↑ in cortex)	[125]
Rg1, *Panax notoginseng* saponins	CUMS-exposed rats	30 mg/kg (*i.p.*) Rg1 for 3 weeks, 100 and 200 mg/kg (*i.p.*) *Panax notoginseng* saponins for 3 weeks	Coat condition↑; Immobility time in FST & TST↓; Time in the open arms↑	Regulation of HPA axis activity (testosterone↓& cortisol↑ in plasma)	[159]
Rg2	CMS-exposed C57BL/6 J mice	10 and 20 mg/kg (*i.p.*) for 2 weeks	SPT↑; Immobility time in FST & TST↓; Weight loss↓	Modulate Neuroplasticity and neurogenesis (p-CREB/CREB, p-TrkB/TrkB, BDNF↑ in hippocampus)	[34]
Rg3	LPS (0.83 mg/kg, *i.p.*)-induced ICR mice	20 and 40 mg/kg (*i.g.*) for 3 days	Immobility time in FST & TST↓; Food intake↑; Weight loss↓	Inhibition of Neural Inflammation (IL-6, IL-1β, Iba-1, p-IκB-α p65/IκB-α p65, p-NF-κB p65/NF-κB p65, Iba-1^+^↓ in hippocampus; IL-6, TNF-α↓ in plasma) Neurotransmitter modulation (Kyn, 5-HIAA, KYN/TRP, 5-HIAA/5-HT, IDO↓ in hippocampus; Kyn, IDO↓ in plasma)	[79]
Rg3	CSDS-exposed C57BL/6 J mice	20 mg/kg (twice/day, *i.p.*) for 3 days	SPT↑; Immobility time in FST & TST↓; Social interaction↑	Modulate Neuroplasticity and neurogenesis (p-CREB/CREB, p-TrkB/TrkB, BDNF↑ in hippocampus)	[24]
Rg3	CMS-exposed C57BL/6 J mice	50, 100 and 150 mg/kg (*i.g.*) for 4 weeks	SPT↑; BW↑; Immobility time in FST & TST↓	Modulate Neuroplasticity and neurogenesis (p-CREB/CREB, BDNF↑ in hippocampus)	[35]
Rg3	CUS-exposed SD rats	10, 20 and 40 mg/kg (*i.g.*) for 2 weeks	Time and enteries in the open arms↑; latency of feed↓	Regulation of HPA axis activity (CORT, CRH, ACTH↓ in serum; progesterone, allopregnanolone↑ in cortex and hippocampus) Neurotransmitter modulation (5-HT↑ in cortex and hippocampus)	[41]
Rg3	CRS-exposed C57BL/6 mice	10, 20 and 40 mg/kg for 2 weeks	SPT↑; Immobility time in FST & TST↓; Total track length in OFT↑	Modulate Neuroplasticity and neurogenesis (Injured cell/Total cell, Iba, PSD-95 puncta/Iba-1 area↓ & cells numbe, the number of dendritic spines, PSD-95, SYN↑ in hippocampal); Inhibition of Neural Inflammation (C1q↓ in peripheral blood and hippocampal)	[151]
Rg5	CSDS-exposed CD1 and C57BL/6 J mice	5, 10, 20 and 40 mg/kg (*i.p.*) for 2 weeks	SPT↑; Immobility time in FST & TST↓; Social interaction↑	Modulate Neuroplasticity and neurogenesis (p-CREB/CREB, p-TrkB/TrkB, BDNF↑ in hippocampus)	[25]
Rh1	CRS-exposed BALB/c mice	10 and 20 mg/kg (*p.o.*) for 2 weeks	Total distance, distance in the central area, dwelling time in the central area↑ time spent and distance traveled in the open arms↑	Inhibition of Neural Inflammation (5-HT↑ in brain; TNF-α, IL-6, CXCL1, adrenaline, CRH, CORT↓ & NE↑ in Serum; IFN-γ and granzyme B levels↑ in CD8 + T and CD4 + T cells in colorectal tumors; IFN-γ levels↑ of CD8 + T and CD4 + T cells in the spleen; MDSC and M-MDSC percentages↓ in the spleen; Populations of MHCII + CD11c + DCs↑ in the spleen, tumor and tumor-draining lymph nodes); Modulation of Gut-Brain Axis (Simpson indices, abundance of Firmicutes↓ & Shannon indices, abundance of Bacteroidota↑ shifts in bacterial composition towards control group)	[160]
Rh2	*T. gondii Fukaya* strain (8 cysts/mouse, *i.g.*) BALB/c mice	50 and 100 mg/kg (*i.g.*) for 4 weeks	Immobility time in FST & TST↓	Inhibition of Neural Inflammation (neuron swollen, neuron shrunken, neuron pyknotic cell bodies, proportion of Annexin V positive neurons, Iba-1, HMGB1, IFN-γ, TNF-α, iNOS, Iba1^+^ cells, proportion of HMGB1 in Iba1 + cells, TLR4, MyD88, TLR4, Iba-1, p-IκB-α/IκB-α, p-NF-κB p65/NF-κB p65, proportion of p-NF-κB p65 in Iba1-positive cells↓& neuron density↑ in cortex)	[20]
Rh2	CUMS-exposed mice	10 and 20 mg/kg (twice/day, *i.p.*) for 4 weeks	SPT↑; Immobility time in TST↓	Modulate Neuroplasticity and neurogenesis (DCX ^+^ cells, p-AKT/AKT, p-CREB/CREB, p-ERK/ERK, BDNF↑ in hippocampus)	[56]
Rh2	Colorectal carcinoma tumor orthotopic implantation Female NOD/SCID mice	0.2, 1 and 5 mg/kg (*i.g.*) for 2 weeks	SPT↑; Immobility time in FST & TST↓; Survival↑	Inhibition of Neural Inflammation (IL-6, IL-18, TNF-α↓ in serum)	[161]
Notoginsenoside R1	CUMS-exposed Wistar rats	50 and 100 mg/kg (*p.o.*) for 1 week	SPT↑; BW↑; Immobility time in FST & TST↓; Grooming, residence duration and rearing↑	Inhibition of Neural Inflammation (TNF-α, IL-6, IL-1β, cleaved caspase-3, Bax, p-NF-κB/NF-κB↓& BDNF, cleaved Bcl-2, p-AKT/AKT, p-PI3K/PI3K, p-NF-κB/NF-κB↑ in hippocampus)	[60]
Rh4	CUMS-exposed C57BL/6 J mice Acute sress depression	5, 10, 20 and 40 mg/kg (*i.g.*) for 1 week or 20 and 40 mg/kg (*i.g.*) for 2 weeks	SPT↑; BW↑; Immobility time in FST & TST↓	Suppression of Oxidative Stress (ROS, 8-OHG, MDA↓ in hippocampus); Modulate Neuroplasticity and neurogenesis (BDNF, Syn, PSD-9, DCX ^+^ cells, spine density of dendrites↑ in hippocampus); Inhibition of Neural Inflammation (TNF-α, IL-6, IFN-γ, IL-1β, GFAP, Iba-1 +, TUNEL + cell density, p-p65, iNOS, COX-2, cleaved caspase-3, cleaved caspase-9↓ in hippocampus; TNF-α, IL-6, IFN-γ, IL-1β↓ in serum) Regulation of HPA axis activity (viable neurons↓ & GR↑ in hippocampus; GR↑ in hypothalamus; CRH, ACTH, CORT↓ in serum)	[57]
Rk1	LPS (0.83 mg/kg, *i.p.*)-induced ICR mice	5, 10 and 20 mg/kg (*i.g.*) for 1 week	Immobility time in FST & TST↓	Modulate Neuroplasticity and neurogenesis (NF-κB, IκB-α↓ and SIRT1, BDNF, TrkB↑in hippocampus); Inhibition of Neural Inflammation (TNF-α, IL-6, IL-1↓ in serum)	[108]
Rk3	CUMS-exposed C57BL/6 J mice	20 and 40 mg/kg (*i.g.*) for 2 weeks	SPT↑; Immobility time in FST & TST↓; Total distance↑	Modulate Neuroplasticity and neurogenesis (BDNF, TrkB, p-ERK, ERK, p-CREB, CREB↑ in cortex; BDNF, PSD-95, Syn, neuronal synapse, astrocytes, microglia↑ in hippocampus; astrocytes, microglia↑ in cortex); Inhibition of Neural Inflammation (IFN-γ, IL-1β, IL-6, TNF-α↓ in hippocampus and cortex); Regulation of HPA axis activity (CRH, ACTH, glucocorticoids↓ in serum) Neurotransmitter modulation (Kyn, 3-HK, Quin, Kyn/Trpcontent↓& Trp, 5-HTP, 5-HT, 5-HTP/Trp↑ in brain, colon, serum; IDO, KMO↓& TPH-2, AAAD↑ in hippocampus, cortex, colon) Modulation of Gut-Brain Axis (IFN-γ, IL-1β, IL-6, TNF-α, intestinal flora disturbance↓ & ZO-1, Occludin, Claudin↑ in colon; acetate, butyrate, propionate, isobutyrate, valerate, caproate↑ in fecal)	[58]
F2	LPS (1 mg/kg, *i.p.*)-induced ICR mice	0.5 and 1 mg/kg (*p.o.*) for 11 days	Immobility time in FST & TST↓	Inhibition of Neural Inflammation (TNF-α, IL-6↓ in serum)	[48]
Compound K	CORT (20 mg/kg, sc.)-induced C57BL/6 J mice	12.5, 25 and 50 mg/kg (*i.p.*) for 21 days	Immobility time in FST & TST↓; Center time in OFT↑	Neurotransmitter modulation (KMO↓ in thalamus)	[152]
Pseudo-ginsenoside HQ (PHQ) Rh2	LPS (0.83 mg/kg, *i.p.*)-induced ICR mice	7.5, 15 and 30 mg/kg (*p.o.*) for 5 weeks	Immobility time in FST & TST↓	Suppression of Oxidative Stress (SOD↑ in hippocampus); Inhibition of Neural Inflammation (TNF-α, IL-6↓ in serum; p-p65/p65, p-p38 MAPK/p38 MAPK, p-NF-κBp65/NF-κBp65, p-NF-IκB-α/NF-IκB-α↓ in hippocampus); Neurotransmitter modulation (R -PHQ: DA, GABA, NE↑ in brain; S -PHQ: DA, GABA↑ in brain)	[84]
Total Ginsenoside Ginseng Root	CUMS-exposed C57BL/6 J mice	40 mg/kg (*i.g.*) for 4 weeks	SPT↑; BW↑; Immobility time in FST & TST↓; Residence time in the target quadrant of Morris water maze↑	Suppression of Oxidative Stress (MDA↑ in serum; ROS↓ in brain; AMPK, SIRT1, PGC-1α, ATP content↓ in hippocampus); Neurotransmitter modulation (5-HT↑ in serum; S -PHQ: DA, GABA↑ in brain)	[61]
Ethanol extract of *P. ginseng*	CUMS-exposed Kunming mice	50, 100 and 200 mg/kg (*i.g.*) for 3 weeks	SPT↑; BW↑; Distance of horizontal movement↑ Times of vertical standing↑	Regulation of HPA axis activity (CRH, ACTH, CORT↓ in serum; KBP51, GR↑& apoptosis neuron↓ in hippocampus)	[141]
Ginsenoside H Dripping Pills	CUMS-exposed SD rats	28, 56 and 112 mg/kg (*i.g.*) for 1 week	SPT↑; Distance of horizontal movement↑ crossing and rearing↑	Modulate Neuroplasticity and neurogenesis (BDNF, p-CREB/CREB, PKA, CAMP↑ in hippocampus); Neurotransmitter modulation (5-HT, DA, NE↑ in hippocampal)	[62]
Kai-xin-san + imipramine	ATCH (100 μg, *i.p.*)-induced Wistar rats	10 mg/kg imipramine (*i.p.*) + 365.4 mg/kg Kai-xin-san (*i.g.*) for 15 days	Immobility time in FST↓; Total distance↑; Standing, duration and entris in center↑	Regulation of HPA axis activity (ACTH, CORT↓ in serum); Neurotransmitter modulation (IDO, KMO, Kyn, quinolinic acid, KYN/TRP, 5-HT2AR↓& KAT, TPH, TRP, 5-HT, KYNA↑ in hippocampus; TDO↓ in liver); Inhibition of Neural Inflammation (CRP, IL-6, TNF-α, IFN-γ, N-methyl-D-aspartate Receptor (NMDAR)↓ & mTOR, p-mTOR, p-mTOR/mTOR, α-amino-3-hydroxy-5-methyl-4-isoxazole propionic acid receptor (AMPAR), BDNF↑ in hippocampal)	[71]
Kai-xin-san + fluoxetine	Fluoxetine-resistant depression Wistar rats	10 mg/kg fluoxetine (*i.g.*) + 491 mg/kg Kai-xin-san (*i.g.*) for 15 days	SPT↑; BW↑; Immobility time in FST↓; Total distance↑; Standing, duration and entris in center↑; Cognitive performance↑	Inhibition of Neural Inflammation (IL-6, TNF-α, IDO-1, TDO-2, KMO, Claudin-5, IBA-1↓ & Neun↑ in hippocampal; TDO-2↓ in liver)	[162]
Xiao-chai-hu-tang	CORT (40 mg/kg, *i.p.*)-induced C57BL/6 J mice	7 g/kg (*i.g.*) for 4 weeks	Immobility time in FST↓	Modulate Neuroplasticity and neurogenesis (neuronal differentiation protein 1↑ in hippocampus)	[163]
Kai-xin-Jie-yu Granule	LPS (0.83 mg/kg, *i.p.*) or CUMS-exposed ICR mice	4000 and 8000 mg/kg (*i.g.*) for 14 days	SPT↑; Immobility time in FST & TST↓; Locomotor activities↑	Inhibition of Neural Inflammation (Nissl bodies↓ & TLR4, p-FOXO1/FOXO1↑ in hippocampal; TNF-α, IL-6, IL-1β↓ in serum)	[63]
Gui-pi-tang	CUMS-exposed SD rats	28, 56 and 112 mg/kg (*p.o.*) for 1 week	SPT↑; Immobility time in FST↓; Latency of feed↓; Time in center↑	Modulate Neuroplasticity and neurogenesis (CORT, neuronal apoptosis, cleaved caspase-3, Bax/Bcl-2↓ & p-CREB/CREB, p-AKT/AKT, p-PI3K/PI3K, BDNF, TrkB↑ in hippocampus)	[72]
Xue-sai-tong soft capsules	Transient middle cerebral artery occlusion + spatial restriction + isolation	0.3, 0.6 and 1.2 g/kg (*p.o.*) for 1 week	SPT↑; Immobility time in TST↓; Total distance, speed and horizontal movement↑	Neurotransmitter Modulation (MAOA↓ & 5-HT, NE, DA↑ in hippocampus; glutamate↓ in cortex and blood)	[73]
Ding-zhi- xiao-wan	LPS (1 mg/kg, i.e.) + CRS-induced ICR mice	668, 1336 and 2672 mg/kg (*p.o.*) for 3 weeks	SPT↑; Immobility time in FST↓; Total distance and speed↑; Entries and time into center area↑	Modulate Neuroplasticity and neurogenesis (p-GluN1/GluN, Iba-1, IL-6, TNF-α, glutamate, nuclear shrinkage, neuron loss, neuronal degeneration↓ & spine density, PSD-95, SYP, mBDNF, p-TrkB, p-ERK1/2, p-mTOR, p-GluA1/GluA1↑ in hippocampus)	[30]
Ban-xia-xie-xin	High-fat ApoE^−/−^ mice	0.45, 1.35 and 4.05 mg/kg (*p.o.*) for 4 weeks	SPT↑; Immobility time in TST↓; Total distance↑	Modulation of Gut-Brain Axis (gut microbiome disorder↓)	[74]

(“↓” and “↑” denote a statistically significant decrease and increase, respectively, *p* < 0.05)

### Animal models of depression-like behaviors

Among experimental models, chronic stress models are the most commonly employed to simulate depressive pathology. The chronic unpredictable mild stress (CUMS) model remains the most widely used in both rats (e.g., Sprague–Dawley [16] and Wistar strains [17]) and mice (e.g., C57BL/6 [18], Kunming [19], and BALB/c strains [20]). CUMS reproduces core symptoms of human depression by repeatedly exposing animals to varied, unpredictable stressors over several weeks, leading to anhedonia and behavioral despair. Variations of the CUMS model, including chronic social defeat stress (CSDS) [18, 21–25], chronic restraint stress (CRS) [26–30], chronic mild stress (CMS) [31–35], chronic unpredictable stress (CUS) [36–41], and unpredictable mild stress (UMS) [42], differ mainly in the type and duration of stressors applied but produce comparable depressive-like phenotypes. Another well‑established method is reserpine‑induced depression, achieved through prolonged administration of low doses of reserpine. This approach induces monoamine depletion, resulting in emotional deficits resembling anhedonia, cognitive impairment, and psychomotor retardation, mirroring key aspects of clinical depression [43]. More recently, glial dysfunction–based models have been introduced to capture structural and cellular abnormalities observed in human depressive disorders. Specifically, intracortical infusion of L‑α‑aminoadipic acid (L‑AAA) into the prefrontal cortex selectively impairs astrocytic function, resulting in both behavioral and histopathological alterations characteristic of depression. Compared with traditional stress models, the L‑AAA paradigm offers greater reproducibility and allows more precise evaluation of therapeutic interventions [44, 45]. For acute depression-like models, lipopolysaccharide (LPS)‑induced models are frequently used to mimic inflammation‑associated depression in both rats (e.g., Wistar [46]) and mice (e.g., C57BL/6 [47] and ICR [48]). Systemic LPS administration triggers an initial phase of sickness behaviors, including fever, anorexia, reduced locomotion, and social withdrawal, typically appearing within approximately 6 h of injection. Depressive‑like behaviors, such as anhedonia and behavioral despair, emerge approximately 24 h after the LPS challenge, reflecting the interplay between inflammatory activation and mood regulation.

### Antidepressant-like effects of ginsenosides

Behavioral paradigms commonly used in depression research include the forced swim test (FST), tail suspension test (TST), and sucrose preference test (SPT) [49]. The FST evaluates behavioral despair, in which animals placed in a water-filled cylinder initially attempt to escape but, with time, adopt an immobile posture; increased immobility duration is interpreted as a marker of depressive‑like behavior [50, 51]. Similarly, the TST measures despair‑like responses by suspending mice by the tail and recording periods of immobility [51]. In contrast, the SPT assesses anhedonia, a core symptom of depression characterized by diminished ability to experience pleasure. Because rodents naturally prefer a sucrose solution over water, reduced sucrose consumption indicates decreased reward sensitivity and serves as an index of anhedonia [52]. Across numerous preclinical studies, a wide variety of ginsenosides and ginseng‑derived herbal formulations have demonstrated significant antidepressant‑like effects in these behavioral tests. Compounds such as ginsenosides Rb1 [53], Rd [54], Re [43, 55], Rg1 [32, 33], Rg2 [34], Rg3 [24], Rg5 [25], Rh2 [56], Rh4 [57], Rk3 [58], 20(*S*)-protopanaxadiol [59], notoginsenoside R1 [60], as well as total ginsenoside extracts [61], Ginsenoside H Dripping Pills [62], and traditional preparations including *Xiao-chai-hu-tang* [40] and *Kai-xin Jie-yu* Granule [63], have shown consistent efficacy. Treatment with these compounds significantly reduced immobility time in the FST and TST and restored sucrose preference in the SPT, strongly supporting their potential to ameliorate depressive‑like behaviors.

### Anxiolytic activities of ginsenosides

Because depression and anxiety frequently coexist and share overlapping neurobiological pathways, anxiety-related behavioral assessments are often employed to complement depressive behavior analyses [64]. These include the open field test (OFT), elevated plus maze test (EPMT), light–dark box test, and novelty-suppressed feeding test (NSFT). In the OFT, rodents are placed in a square arena, and parameters such as locomotor activity, rearing, and grooming are recorded. A preference for the periphery (thigmotaxis) indicates anxiety-like behavior, whereas greater exploration of the central area reflects reduced anxiety [51, 52, 65]. In the EPMT, which comprises two open and two closed arms elevated above the floor, avoidance of open arms represents anxiety-like behavior, while increased time and crossings in open arms indicate anxiolytic or antidepressant-like effects [66]. In the light–dark box test, anxious animals tend to remain in the dark area; lower anxiety levels are reflected by shorter latency to enter, and longer duration spent in, the illuminated zone [52]. The NSFT, on the other hand, assesses motivational and emotional reactivity to novel environments by measuring the latency to feed and the amount of food consumed after stress exposure [67]. Several ginsenosides and ginseng-containing formulations have been shown to enhance performance across these paradigms. Compounds such as ginsenosides Rb1 [68], Rd [69], Re [55], Rg1 [70], Rk3 [58], 20(*S*)-protopanaxadiol [59], notoginsenoside R1 [60], Ginsenoside H Dripping Pills [62], *Kai-xin-san* [71], *Gui-pi-tang* [72], *Xue-sai-tong* soft capsules [73], *Ding-zhi-xiao-wan* [30], and *Ban-xia-xie-xin-tang* [74], have improved exploratory behavior in the OFT. In the EPMT, combinations such as Rb1 with berberine [16], as well as Rg1 [29], Rg3 [41], and *Bifidobacteria*-fermented red ginseng (fRG) [75], increased open-arm exploration and reduced avoidance behavior. Both regular and fermented ginseng preparations also enhanced performance in the light–dark box test, while Rg1 [39], Rg3 [41], and *Gui-pi-tang* [72] also showed significant improvements in the NSFT.

Together, the findings across these behavioral paradigms provide robust preclinical evidence that ginsenosides exert antidepressant and anxiolytic activities through multifaceted mechanisms. These outcomes reinforce the potential of ginsenosides as multifunctional neuroactive compounds with promising applications in mood regulation and psychotherapy development.

## Underlying mechanisms of the antidepressant activities of ginsenosides

The molecular mechanisms underlying the antidepressant-like effects of ginsenosides are multifaceted and involve the regulation of multiple interconnected biological systems associated with depression. These systems include neurotransmission, oxidative stress, neuroinflammation, neuroplasticity and neurogenesis, HPA axis function, and the gut–brain axis. The representative molecular pathways and targets implicated in these processes are summarized in Fig. 2.

### Neurotransmitter modulation

Disturbances in neurotransmitter systems play a central role in the pathophysiology of major depressive disorder (MDD) [76–78]. Dysregulation within serotonergic, dopaminergic, and GABAergic pathways significantly disrupts neural circuits that regulate mood, cognition, motivation, and stress responses. Notably, ginsenosides have demonstrated the capacity to restore this equilibrium by modulating multiple facets of neurotransmitter metabolism and receptor signaling.

#### Modulation of serotonergic system

Evidence from stress‑induced models indicates that ginsenosides effectively normalize serotonergic transmission. The serotonin (5-HT) system plays a pivotal role in mood regulation and antidepressant responses Treatment with 20(*S*)‑protopanaxadiol or total ginsenosides extracted from *Panax ginseng* significantly increases hippocampal and cortical 5‑HT levels suppressed by CUMS in mice [59, 61]. Ginsenoside Rg3 prevents CUS‑induced reductions of 5‑HT levels in the prefrontal cortex and hippocampus, suggesting that its antidepressant-like effects are closely associated with the stabilization of serotonergic signaling [41]. Similarly, Rb1 elevates hippocampal concentrations of 5‑HT and tryptophan (Trp) while reducing kynurenine (Kyn) levels and IDO activity, accompanied by enhanced expression of postsynaptic 5‑HT₁A receptors. These findings indicate that Rb1 alleviates depressive‑like behaviors by correcting monoaminergic imbalances and reinforcing serotonergic receptor function [48].

Pretreatment with Rb1 further elevates hippocampal levels of 5‑HT, its metabolite 5-HIAA, as well as norepinephrine (NE) and dopamine (DA) in CUMS-exposed rats [17]. Formulations containing ginsenosides, such as Ginsenoside H Dripping Pills and *Xue-sai-tong* soft capsules, also enhance central 5‑HT, NE, and DA levels in both stress‑induced [62] and ischemia‑induced [73] depression models. Under inflammatory conditions induced by LPS, Rg3 lowers hippocampal Kyn and 5‑HIAA contents, attenuates IDO activity, and normalizes both Kyn/Trp and 5‑HIAA/5‑HT ratios, evidencing restored serotonergic homeostasis [79]. Additionally, maternal *Toxoplasma gondii* infection during pregnancy impairs serotonergic and dopaminergic signaling by increasing IDO expression and decreasing tyrosine hydroxylase (TH) levels; treatment with Rh2 reverses these effects in mouse offspring, restoring normal 5‑HT and DA synthesis in the prefrontal cortex [20].

#### Regulation of tryptophan metabolism

Ginsenosides also modulate peripheral Trp metabolism an essential contributor to the serotonergic–Kyn balance associated with mood regulation. Dysregulated Trp metabolism contributes to depression through (i) reduced 5-HT biosynthesis due to Trp depletion and (ii) overactivation of the Kyn pathway, which generates neurotoxic intermediates [76]. The enzymes indoleamine-2,3-dioxygenase (IDO) and tryptophan-2,3-dioxygenase (TDO) catalyze the initial step of Trp catabolism to Kyn, while in the serotonergic route, monoamine oxidase (MAO) degrades 5-HT into 5-hydroxyindoleacetic acid (5-HIAA). Imbalances within these pathways result in neurotransmitter dysregulation [80, 81]. Under CUMS, mice typically exhibit reduced levels of Trp, 5‑hydroxytryptophan (5‑HTP), and 5‑HT, accompanied by elevated concentrations of Kyn, 3‑hydroxykynurenine (3‑HK), and quinolinic acid (Quin) [58]. Ginsenoside Rk3 reverses these abnormalities by normalizing the Kyn/Trp and 5‑HTP/Trp ratios, thereby shifting Trp metabolism toward 5‑HT synthesis. Mechanistically, Rk3 downregulates IDO and Kyn monooxygenase (KMO), while upregulating tryptophan hydroxylase (TPH-1/2), aromatic L-amino acid decarboxylase (AAAD), and kynurenine aminotransferase (KAT) Similar enzyme expression changes are observed in the colon, emphasizing the significance of gut–brain metabolic integration in the antidepressant-like effects of ginsenosides [58]. Ginsenoside Rg1 also reduces Kyn accumulation in the serum, hippocampus, and liver by enhancing hepatic expression of KAT2 and KMO, which promote the detoxification of Kyn metabolites. These actions suggest that Rg1 mitigates depressive-like symptoms by promoting peripheral Kyn metabolism and minimizing the neurotoxic burden on the brain [33]. Traditional formulations containing ginsenosides show comparable activity. The classical herbal medicine *Kai-xin-san*, administered alone or in combination with imipramine, enhances TPH and KAT enzymatic activity, elevates 5-HT and kynurenic acid concentrations, and decreases TDO, KMO, and Quin levels in both the hippocampus and liver [71].

#### Dopaminergic and noradrenergic regulation

Beyond serotonergic regulation, ginsenosides also influence catecholaminergic transmission. Ginsenosides such as Rg3, Rb3, and compound K decrease excessive NE levels in the frontal cortex and hippocampus, reflecting the normalization of stress-induced hyperactivity within noradrenergic circuits [82]. In behavioral despair models, treatment with Rb1 restores hippocampal and cortical levels of 5‑HT, 5‑HIAA, NE, DA, and γ-aminobutyric acid (GABA), while simultaneously lowering excess glutamate in the CA3 region and prefrontal cortex. These results suggest that Rb1 exerts combined modulatory effects on monoaminergic and amino acid neurotransmission [17, 83]. The stereochemistry of ginsenosides also contributes to their neurochemical specificity. For example, the *R*-isomer of pseudo-ginsenoside HQ modulates dopaminergic, GABAergic, and noradrenergic systems, whereas the S-isomer predominantly affects dopaminergic and GABAergic pathways in ICR mice [84]. Such stereochemical distinctions underscore the structure–activity sensitivity of ginsenoside interactions within neurotransmitter networks.

#### Glutamatergic and GABAergic regulation

Glutamate, the principal excitatory neurotransmitter in the CNS, is essential for synaptic plasticity, cognition, and adaptive stress responses [78, 85]. Within the glutamate–glutamine cycle [86], astrocytic glutamine synthetase catalyzes the conversion of glutamate to glutamine, while neuronal glutamate decarboxylase converts glutamate to GABA, the major inhibitory neurotransmitter [87, 88]. Glutamate also serves as a precursor for glutathione, a critical antioxidant protecting neurons from oxidative damage [89]. Thus, disruption in glutamate–GABA balance impairs both neurotransmission and neuronal resilience. Ginsenosides such as Rb1 and pseudo-ginsenoside HQ elevate cerebral GABA concentrations, contributing to the restoration of the excitatory–inhibitory equilibrium essential for emotional stability [83, 84]. By modulating both glutamatergic and GABAergic signaling, these compounds stabilize synaptic communication and mitigate neuronal hyperexcitability commonly associated with depressive phenotypes.

Collectively, ginsenosides exert broad neurochemical regulatory actions across multiple neurotransmitter systems. By rebalancing tryptophan metabolism, restoring serotonergic and catecholaminergic function, and stabilizing glutamate–GABA interplay. These natural compounds target the molecular foundations of depression. This multimodal modulation of neurotransmission provides a strong mechanistic basis for their amelioration of stress‑related observed in diverse experimental models.

### Inhibition of neural inflammation

#### Neuroinflammatory pathways in depression

**Fig. 3:**
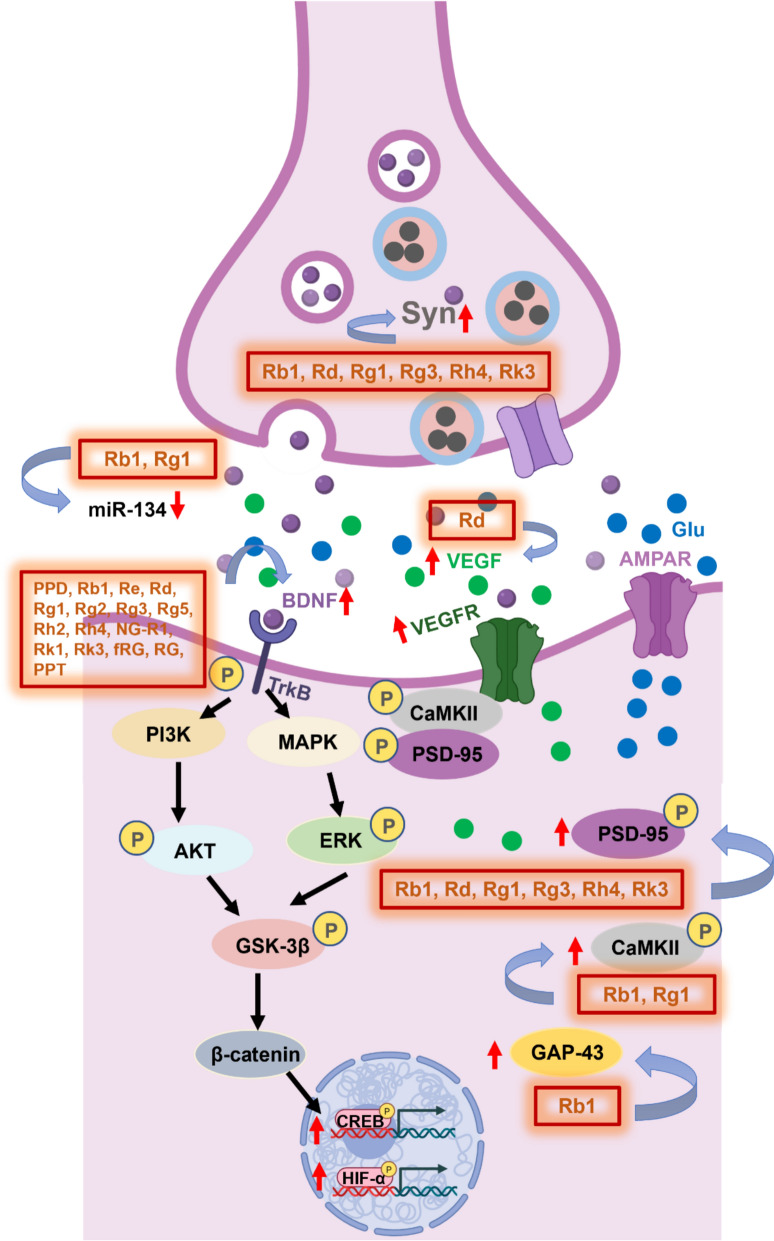
Mechanistic insights into ginsenoside-induced neuroplasticity and neurogenesis

Patients with clinical and subclinical depression frequently exhibit elevated levels of peripheral inflammatory markers [90]. Mounting evidence indicates that inflammation is a central driver of depressive pathophysiology and that modulating neuroinflammatory signaling may attenuate disease progression and symptom severity [91–93]. Cytokines are key mediators of inflammation and are released by a wide variety of cell types [94, 95]. Within the CNS, microglia, astrocytes, and neurons are primary sources of pro‑inflammatory cytokines [96]. The nuclear factor‑κB (NF‑κB) pathway is a major transcriptional regulator of cytokine gene expression [97]. NF‑κB typically comprises the p65 and p50 subunits [98] and remains sequestered in the cytoplasm under resting conditions by binding to inhibitory IκB proteins [97]. Upon stimulation, the pathway induces IκB phosphorylation and degradation, releasing active NF‑κB dimers to the nucleus [99]. Various pro‑inflammatory stimuli, such as tumor necrosis factor‑α (TNF‑α), interleukin‑1 (IL‑1), high‑mobility group box 1 (HMGB1), and interferon‑γ (IFN‑γ), activate NF‑κB via receptors including tumor necrosis factor receptor (TNFR), IL‑1R, and toll-like receptor 4 (TLR4) [100, 101]. Among these, IL‑1β plays a pivotal role by amplifying cytokine and chemokine production [102]. Its activation of IL‑1β requires inflammasome assembly, particularly the NLRP3 inflammasome, which contains NLRP3, the adaptor apoptosis-associated speck-like protein (ASC), and procaspase‑1 [103, 104]. TLR4‑mediated signaling, often triggered by LPS, recruits the adaptor myeloid differentiation primary response 88 (MyD88), leading to NF‑κB activation and the subsequent induction of inflammatory mediators such as TNF‑α, IL‑1, IL‑6, and IL‑18, as well as chemokines (CXCL10, CCL2, CCL3), adhesion molecules (CD11b, CD45), and enzymes, such as inducible nitric oxide synthase (iNOS), and COX‑2 [105]. The mechanistic basis of ginsenoside‑induced suppression of neuroinflammation is summarized in Fig. 3.

#### Anti‑neuroinflammatory actions of ginsenosides

A substantial body of studies demonstrates that ginsenosides suppress neuroinflammatory signaling and consequently mitigate depressive‑like behaviors. Ginsenoside Rb1 markedly reduced hippocampal TNF‑α and IL‑6 levels and inhibited the activation of Ras–mitogen-activated protein kinase (MAPK)/NF‑κB signaling [48]. In CRS models, Rb1 downregulated hippocampal TLR4/NF‑κB/C3 signaling and decreased astrocyte and microglia activation, promoting a shift toward an anti‑inflammatory microglial phenotype and preventing excessive synaptic pruning [27]. During neuroinflammation, astrocyte‑derived complement C3 is cleaved into C3a, which binds C3aR on neurons and microglia, amplifying injury and inflammatory responses [27, 106, 107]. Dong‑Hyun Kim and colleagues reported that environmental stress elevated NF‑κB activation, IL‑6 production, and corticosterone (CORT) levels while increasing NF‑κB⁺/Iba1⁺ microglia in the hippocampus. Treatment with red ginseng (RG) or fRG reversed these effects, suppressed neuroinflammation, and alleviated anxiety‑ and depression‑like behaviors. RG and fRG also attenuated colitis by downregulating NF‑κB activation and associated inflammatory markers, suggesting brain–gut axis coordination in their anti‑inflammatory effects [75]. Rg1 inhibited the chronic stress‑induced elevation of IL‑1β by blocking NF‑κB activation and reducing NLRP3 inflammasome expression  [70]. Similarly, Rg3 attenuated both hippocampal and peripheral levels of IL‑1β, IL‑6, and TNF‑α in LPS‑induced neuroinflammation, decreased phosphorylated IκBα and p65 expressions, and limited microglial overactivation [79]. In another study, Rk1 also prevented LPS‑induced elevation in p‑NF‑κB and p‑IκBα by enhancing sirtuin-1 (SIRT1) expression, a NAD‑dependent deacetylase that inhibits NF‑κB signaling and provides neuroprotection [108, 109].

Consistently, Rh2 and pseudo‑ginsenoside HQ (*S*- and *R*- forms) suppressed NF‑κB and IκBα phosphorylation in brain tissues, resulting in dose‑dependent reductions of TNF‑α and IL‑6 levels. These anti‑inflammatory actions likely underlie their amelioration of stress‑related [84]. Rh2 additionally reduced HMGB1, TLR4, and phosphorylated NF‑κB p65 expression in the prefrontal cortex of offspring exposed to maternal *Toxoplasma gondii* infection [20]. By modulating the HMGB1/TLR4/NF‑κB pathway, Rh2 alleviated neuroinflammation and improved behavioral outcomes. HMGB1, a chromatin‑binding protein, serves as a DAMP that activates innate immune signaling via TLR4 signaling [110–112].

Other ginsenosides act through additional molecular cascades. Notoginsenoside R1 modulates phosphatidylinositol 3-kinase (PI3K)/protein kinase B (Akt)/NF‑κB p65 protein levels in chronic mild stress models, suggesting involvement of the PI3K/Akt/NF‑κB axis in its antidepressant mechanism [60]. Rb1 suppresses the release of TNF‑α, L‑18 and IL‑1β, limits microglial activation, and upregulates antioxidant mediators Nrf2, HO‑1, and SIRT1 [21]. Through the SIRT1–NLRP3/Nrf2 pathway, Rb1 exerts both anti‑inflammatory and neuroprotective effects. Additional studies demonstrate that Rb1 enhances Akt activation, suppresses hippocampal and serum IL‑1β and TNF‑α, and reduces cytokine production in LPS‑stimulated microglia, firmly linking its antidepressant benefits to inhibition of hippocampal and systemic inflammation [26]. Further reinforcing these findings, Rg1 consistently reduced Iba1‑positive microglia and cytokine levels in the hippocampus [21], inhibited inflammation by decreasing connexin 43 ubiquitination [36], exhibited robust anti‑inflammatory activity across multiple depression models [22, 23, 47, 70, 113]. Ginsenoside F2 significantly decreased serum levels of TNF‑α and IL‑6 in LPS‑induced acute depression models [48]. Traditional ginseng‑containing formulations, such as *Kai-xin-san* and *Xiao-chai-hu-tang*, recapitulate these anti‑inflammatory effects, demonstrating that the antidepressant potential of ginsenosides is closely linked to their suppression of neuroimmune activation [30, 63, 71].

### Suppression of oxidative stress

Oxidative stress and inflammation are interdependent processes that reinforce one another in the pathophysiology of depression [114]. Excessive oxidative stress promotes neuronal dysfunction, disrupts synaptic plasticity, and contributes to volume reductions in the frontal cortex and hippocampus—structural abnormalities frequently observed in depressive disorders [115]. Reactive oxygen species (ROS) activate intracellular signaling cascades that stimulate pro-inflammatory gene expression, while activated immune cells further generate ROS and inflammatory mediators (e.g., cytokines, chemokines), amplifying tissue injury at inflammatory sites [116].

An imbalance between ROS production and antioxidant defense impairs redox homeostasis, leading to neuronal damage and altered neurotransmission [117]. Although the causal link between ROS and depression remains under investigation, patients with depressive symptoms display consistently elevated oxidative stress markers [118]. Lipid peroxidation, a hallmark of oxidative stress, occurs when ROS attack membrane phospholipids [119], which is particularly deleterious in the brain [120]. Malondialdehyde (MDA), a terminal product of lipid peroxidation, is widely recognized as a biochemical biomarker of depression [121]. To counteract ROS-induced injury, cells activate antioxidant defense pathways that upregulate enzymes such as heme oxygenase-1 (HO-1), glutathione peroxidase (GPx), superoxide dismutase (SOD), and catalase (CAT) [122, 123]. Together, these systems preserve redox equilibrium and safeguard neuronal integrity [124].

Experimental studies have shown that ginsenosides alleviate oxidative stress and contribute to antidepressant-like effects by reestablishing redox homeostasis. In CUMS models, oral administration of 20(*S*)-protopanaxadiol markedly reduced serum MDA levels and alleviated oxidative injury, indicating a close association between antioxidative action and amelioration of stress‑related [59]. Likewise, ginsenoside Re normalized hippocampal MDA concentrations and restored SOD activity in reserpine-treated mice, demonstrating improved oxidative resilience [43]. Ginsenoside Rg1 also exhibits particularly robust antioxidant capacity. In CUMS-induced depressive states, Rg1 reduced markers of lipid and DNA oxidation, including MDA, nitric oxide (NO), 4-hydroxynonenal (4-HNE), MitoSOX, and 8-hydroxy-2’-deoxyguanosine (8-OHdG), while enhancing SOD and GPx activity and regulating NADPH oxidase (NOX1/NOX4) expression in the hippocampal CA1 region [113]. Consistent results were observed in models of prenatal methimazole exposure, where Rg1 corrected dysregulated MDA, SOD, CAT, and GPx levels in both mothers and offspring, alleviating postpartum depressive behavior and improving motor function [125]. Other ginsenosides demonstrate comparable antioxidative actions. Rh4 attenuated ROS production and normalized 8-OHdG and MDA levels in CUMS mice [57], while Rk1 stabilized MDA and SOD levels in LPS-exposed brains, thereby preventing depression-like phenotypes [108]. Total ginsenoside extracts from *Panax ginseng* roots also restored plasma MDA levels in stress-induced depressive mice, supporting systemic antioxidant benefits [61].

In CSDS models, ginsenoside Rb1 enhanced SOD and CAT activity, reduced lipid peroxidation, and activated the Nrf2/HO-1 pathway, a key antioxidant axis that maintains redox homeostasis and protects against neuronal injury [21]. Similarly, in LPS-treated mice, Rh2, pseudo-ginsenoside HQ (*R*- and S- forms) restored hippocampal SOD activity and reversed downregulation of SIRT1. While Rh2 modestly influenced Nrf2 expression, both pseudo-ginsenoside isomers produced stronger Nrf2 activation, implying compound-specific modulation of hippocampal redox-signaling [84].

Collectively, these findings indicate that ginsenosides mitigate oxidative stress–related depression by enhancing antioxidant enzyme activity, regulating redox‑sensitive signaling pathways such as Nrf2/HO‑1 and SIRT1, and protecting neuronal integrity. Through these integrated actions, ginsenosides maintain neuronal redox stability, reduce oxidative-inflammatory feedback loops, and thereby contribute to their broad neuroprotective and amelioration of stress‑related.

### Modulate neuroplasticity and neurogenesis

**Fig. 4:**
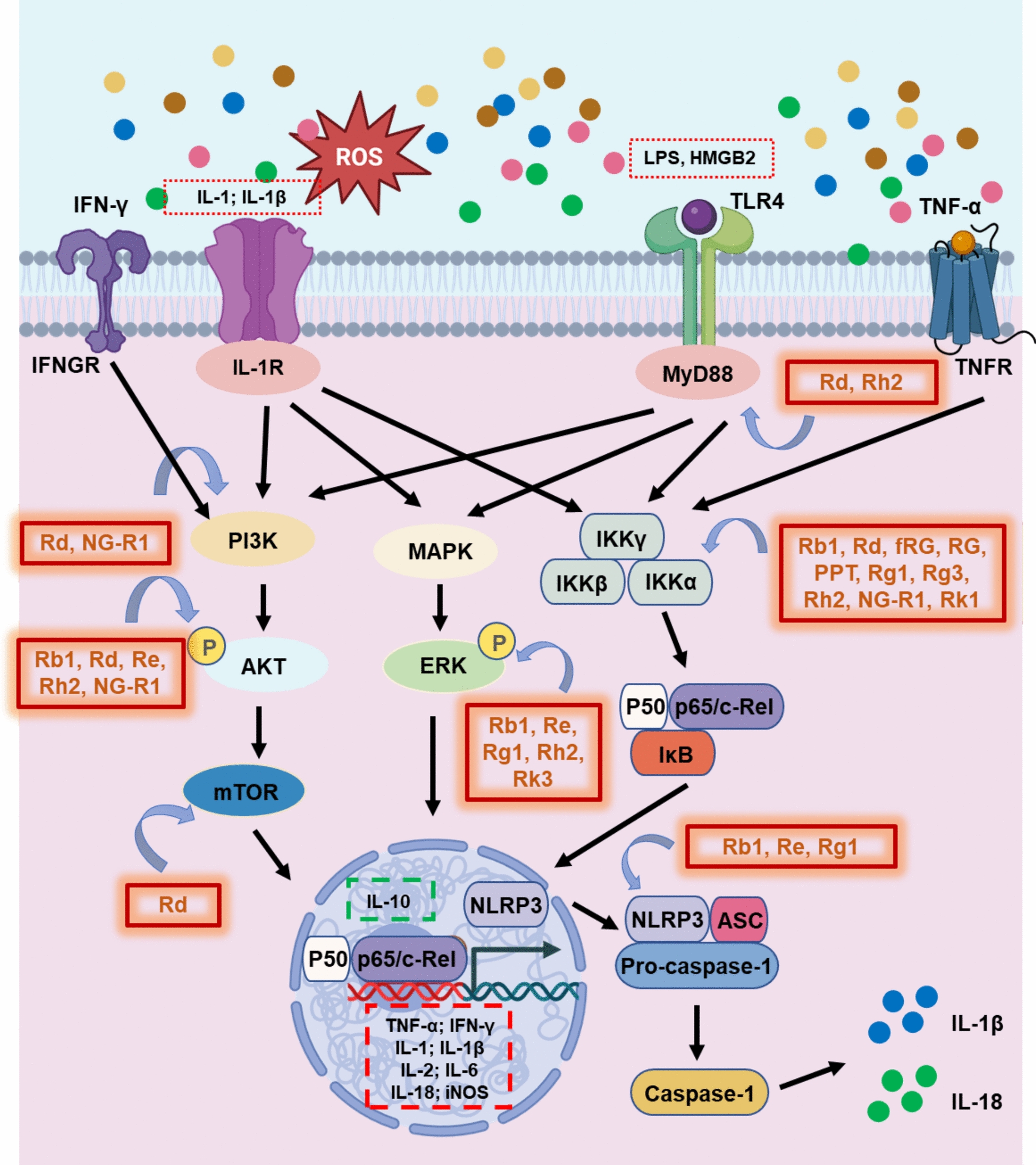
Mechanistic basis of ginsenoside-mediated suppression of neuroinflammation

Depression-like behaviors are strongly associated with impairments in neuroplasticity and synaptic remodeling. At the cellular level, chronic stress leads to neuronal atrophy and synaptic loss, particularly within the prefrontal cortex and hippocampus, the regions critical for emotion regulation and cognitive processing. Among the neurotrophic mechanisms implicated, brain‑derived neurotrophic factor (BDNF) signaling plays a pivotal role in maintaining neural structure and function [126]. Decreased BDNF expression correlates with hippocampal volume reduction and emotional dysregulation in depressive disorders [127]. BDNF exerts its neurotrophic effects primarily through activation of the tropomyosin receptor kinase B (TrkB) receptor [128]. Upon BDNF binding, TrkB undergoes autophosphorylation, triggering downstream signaling cascades—including phospholipase C-γ (PLC-γ), MAPK/extracellular signal-regulated kinase (ERK), and PI3K/AKT—that converge on cAMP response element–binding protein (CREB), a transcription factor essential for BDNF synthesis, synaptic maintenance, and neuroprotection [129]. Activated CREB reinforces its own signaling via a positive feedback loop that enhances endogenous BDNF production [130], thereby sustaining structural neuroplasticity. Within this framework, proteins such as TrkB, AKT, ERK, GSK-3β, β-catenin, and CREB serve as key molecular indicators of synaptic integrity and neurogenesis [131]. The mechanistic insights into ginsenoside‑driven neuroplasticity and neurogenesis are summarized in Fig. 4.

Accumulating evidence reveals that ginsenosides enhance neuroplasticity and neurogenesis, contributing critically to their antidepressant potential. Under chronic stress, synaptic loss and dendritic retraction occur prominently in the hippocampus and prefrontal cortex; restoration of these structures is essential for behavioral recovery [46, 53, 54].

Ginsenoside Rb1 promotes hippocampal synaptic plasticity in CUMS-exposed mice [53]. It increases dendritic spine density in the CA1, CA3, and dentate gyrus regions and improves postsynaptic architecture, manifested as elongated and thickened postsynaptic densities (PSDs) with reduced synaptic cleft width [53]. At the molecular level, Rb1 upregulates BDNF and its downstream mediators (TrkB, AKT, ERK1/2, GSK-3β, β-catenin, and CREB) while downregulating microRNA-134 (miR-134), which binds the 3′UTR of BDNF mRNA and suppresses its translation. Inhibiting miR-134 thus enhances BDNF expression and facilitates synaptic remodeling [53].

MicroRNAs (miRNAs), the short, conserved non-coding RNAs, regulate nearly half of human gene expression [132]. The brain-enriched miR-134 modulates activity-dependent dendritic growth and synaptic plasticity [53, 132]. Therefore, suppression of miR-134 by Rb1 provides a molecular link between ginsenoside action and enhanced neuronal connectivity. Consistently, Rb1 treatment reversed CSDS-induced impairments in BDNF signaling and hippocampal neurogenesis. It restored the phosphorylation of ERK, AKT, and CREB, and increased the number of doublecortin-positive (DCX⁺) cells in the dentate gyrus, indicating activation of BDNF-dependent neurogenic pathways [18].

Other ginsenosides exhibit similar effects. Treatment with RG and fRG significantly increased BDNF expression and expanded the population of BDNF⁺/NeuN⁺ hippocampal neurons [75]. Ginsenoside Re reversed oxidative stress- and reserpine-induced suppressions of the BDNF/TrkB/ERK/CREB pathway both in HT-22 neuronal cells and in the mouse hippocampus, confirming its neuroprotective and antidepressant properties via reactivation of this key cascade [43].

Vascular endothelial growth factor (VEGF), a well-characterized angiogenic cytokine, binds to VEGFR-1 and VEGFR-2 receptors within the CNS to regulate neurogenesis, neuronal survival, and vascular repair [133–135]^.^ Activation of these receptors stimulates downstream signaling cascades, including MAPK/ERK, PI3K/AKT, and PLC-γ/MAPK, which promote neuronal differentiation, synaptic recovery, and structural neuroplasticity, the processes that align closely with the neurorestorative effects observed in ginsenoside-treated models. Among these compounds, ginsenoside Rd has been shown to promote neuronal growth and angiogenesis primarily via the VEGF-dependent PI3K/AKT/mTOR pathway [69]. In CMS models, Rd restored hippocampal levels of synapsin I (SYN1) and PSD-95, both essential for synaptic integrity, and upregulated VEGFR-2, PI3K, AKT, and mTOR expression at both mRNA and protein levels. These effects appear to be mediated through activation of the HIF-1α/VEGF axis and VEGFR-2-dependent PI3K/AKT/mTOR signaling, which collectively support synaptic remodeling, neuronal regeneration, and angiogenic maintenance [69].

Collectively, findings from diverse experimental models indicate that ginsenosides enhance neuroplasticity and neurogenesis through coordinated activation of neurotrophic (BDNF/TrkB/CREB) and angiogenic (VEGF/PI3K/AKT/mTOR) pathways. By restoring synaptic structure, dendritic morphology, and trophic signaling, ginsenosides promote neuronal resilience and functional recovery—mechanisms fundamentally linked to their antidepressant-like effects.

### Regulation of HPA axis activity

The HPA axis is the central neuroendocrine system orchestrating the body’s stress response by controlling the release of adrenocorticotropic hormone (ACTH) and cortisol [136]. In response to physiological or psychological stress, the hypothalamus secretes corticotropin-releasing hormone (CRH), which stimulates the anterior pituitary to release ACTH. ACTH then acts on the adrenal cortex to produce glucocorticoids (e.g., cortisol and CORT), the terminal effectors of the HPA axis that mediate stress adaptation [137–139]. Glucocorticoids exert their effects primarily through the nuclear glucocorticoid receptor (GR), a ligand-activated transcription factor that maintains emotional and metabolic homeostasis under normal conditions [140]. However, dysregulation of the HPA axis is a hallmark of depressive disorders, often manifesting as chronic hypercortisolism and impaired glucocorticoid feedback inhibition, both of which contribute to neuronal damage, synaptic dysfunction, and emotional instability.

Numerous studies demonstrate that ginsenosides can restore HPA axis balance, thereby contributing to their amelioration of stress‑related. For instance, co-administration of berberine and ginsenoside Rb1 significantly alleviated depressive-like behaviors in diabetic rats, while reducing stress-induced elevations in plasma cortisol and ACTH, suggesting normalization of neuroendocrine function [16]. Similarly, Rg3 treatment decreased serum ACTH and CORT levels in mice, indicating effective suppression of endocrine hyperactivation associated with chronic stress [82]. Consistent results were also observed in other experimental models utilizing herbal formulations. The herbal formula *Kai-xin-san*, as well as its combination with imipramine, markedly lowered serum ACTH and CORT levels in ACTH-induced depressive rats, reflecting a synergistic modulation of the HPA axis [71].

In another study, Rg3 inhibited chronic stress-induced increases in CRH, ACTH, and CORT levels in Sprague–Dawley rats, linking its anxiolytic-like actions to restoration of endocrine homeostasis [41]. Ginsenosides Rh4 and Rk3 produced similar outcomes in CUMS-exposed mice, normalizing serum CRH, ACTH, and CORT concentrations while enhancing GR protein expression, suggesting increased glucocorticoid sensitivity and improved negative feedback regulation [57, 58]. Consistent findings by Wu et al. showed that an ethanol extract of *Panax ginseng* roots ameliorated depressive-like behaviors in CUMS mice by lowering CRH, ACTH, and CORT levels. This effect was accompanied by suppression of FKBP51, a chaperone protein that inhibits GR activity, thereby enhancing GR signaling and stabilizing stress hormone feedback [141].

Collectively, these findings indicate that ginsenosides restore HPA axis homeostasis through multiple convergent mechanisms, including reducing circulating glucocorticoid levels, enhancing GR expression and sensitivity, and suppressing stress‑induced endocrine overactivation [63, 70, 75]. This normalization of neuroendocrine function likely contributes substantially to the antidepressant and stress‑protective effects of ginsenosides.

### Modulation of gut-brain axis

Emerging evidence underscores a bidirectional communication between the gut microbiota and CNS, a relationship increasingly recognized in the pathogenesis of depressive disorders [142]. Gut dysbiosis, or disruption of the intestinal microbial community, has been consistently associated with altered immune and neuroimmune function, leading to physiological, metabolic, and behavioral abnormalities [143]. Such imbalance perturbs the microbiota-gut-brain axis, influencing mood regulation through multiple mechanisms, including modulation of neurotransmitter synthesis, production of microbial metabolites (e.g., short-chain fatty acids and *D*-amino acids), and mediation of systemic inflammatory signaling [144, 145]. These insights identify the gut microbiota as a promising therapeutic target for mitigating depressive symptoms and enhancing amelioration of stress‑related.

Several studies demonstrate that ginsenosides can rebalance gut microbial communities while producing antidepressant and anxiolytic effects. Treatment with RG and fRG ameliorated *Escherichia coli* K1 (EC)-induced gut dysbiosis by increasing beneficial *Bacteroidetes* populations and reducing *Proteobacteria* abundance, taxonomic shifts often associated with stress-related inflammation. Notably, fRG and its active metabolites, Rd and protopanaxatriol, elicited robust antidepressant- and anxiolytic-like effects, closely linked to the restoration of microbial balance [75]. Similarly, the herbal preparation *Ban-xia-xie-xin* improved atherosclerosis-associated depression by normalizing gut microbiota composition and regulating lipid metabolism across both central and peripheral systems [74]. Complementary findings show that ginsenoside Rk3 alleviated CUMS-induced dysbiosis by restructuring microbial communities, increasing short-chain fatty acid (SCFA) production, and upregulating tight-junction proteins essential for maintaining intestinal barrier integrity. These effects were accompanied by reduced inflammatory cytokine levels, collectively contributing to the compound’s antidepressant-like activity [58].

Collectively, these studies suggest that ginsenosides exert mood-stabilizing and neuroprotective benefits not only through central neurochemical modulation but also via peripheral mechanisms that restore gut microbial equilibrium, enhance intestinal barrier function, and attenuate systemic inflammation. By strengthening the microbiota–gut–brain axis, ginsenosides contribute to the integrated regulation of neuroimmune homeostasis, forming a vital peripheral pathway underlying their amelioration of stress‑related.

### Integration of ginsenoside actions along the pathophysiological cascade of depression

Although the antidepressant-related mechanisms of ginsenosides are often described in discrete categories, accumulating evidence indicates that these pathways are functionally interconnected rather than operating in isolation. Depression is increasingly conceptualized as a disorder driven by a pathological cascade, in which chronic stress induces dysregulation of the HPA axis, followed by disturbances in monoaminergic neurotransmission [146], activation of neuroinflammatory [147] and oxidative stress pathways [148], impairment of neuroplasticity [149], and disruption of the gut–brain axis [150].

Within this cascade-based framework, different classes of ginsenosides appear to preferentially modulate distinct pathological layers rather than acting uniformly across all mechanisms. Dammarane-type ginsenosides, particularly PPD and PPT derivatives such as Rb1, Rg1, and Re, consistently exhibit broad, multi-target activities involving neurotransmitter regulation, neurotrophic signaling (BDNF/CREB), and normalization of HPA axis function. These compounds predominantly act on central neurochemical and neuroplastic nodes, thereby supporting structural and functional recovery under chronic stress conditions [18, 21, 26, 28, 32, 36, 37, 43, 47, 48, 53, 55, 68]

In contrast, rare ginsenosides generated through deglycosylation (e.g., Rg3, Rg5, Rk1, and compound K), which generally display enhanced bioavailability, demonstrate comparatively stronger modulatory effects on inflammation- and oxidative stress–related pathways, including NF-κB signaling, NLRP3 inflammasome activation, and redox-sensitive cascades. and stronger potency toward inflammation- and oxidative stress–related pathways, including NF-κB, NLRP3 inflammasome activation, and redox-sensitive signaling. Their preferential activity at immune–metabolic and inflammatory interfaces suggests a complementary role in regulating downstream effectors of depressive pathology [25, 108, 151, 152].

Importantly, these structure-associated tendencies imply potential synergistic or additive interactions among ginsenosides acting at different hierarchical levels of the depressive cascade. Multi-component ginsenoside compositions or sequential biotransformation products may collectively stabilize upstream neuroendocrine disturbances while simultaneously attenuating downstream neuroimmune and oxidative damage. Although pharmacodynamic synergy has not been conclusively demonstrated, convergent evidence from preclinical models supports a systems-level paradigm in which ginsenosides exert antidepressant-like effects through coordinated, multilayer modulation rather than isolated single-target actions.

Taking together, current evidence supports a hierarchical, system-oriented model in which ginsenosides modulate interconnected pathological processes underlying depression. This integrative perspective provides a more coherent framework for understanding their multitarget pharmacology and may inform the rational design of future combination strategies and translational investigations.

## Conclusions and future perspectives

In recent years, there has been increasing interest in the development of ginsenosides as a novel therapeutic approach for depressive disorders. In this review, we summarized and synthesized the reported biological functions of ginsenosides in relation to their antidepressant potential. As illustrated in Fig. 2, the mechanisms underlying the antidepressant-like effects of ginsenosides and ginsenoside‑containing herbal formulations involve multiple interrelated pathways, including the suppression of oxidative stress, inhibition of neuroinflammation, enhancement of neuroplasticity and neurogenesis, and regulation of HPA axis activity. Collectively, these findings suggest that ginsenosides are promising candidates for the prevention and improvement of depression‑like behaviors.

Despite substantial progress, current research on the antidepressant-like effects of ginsenosides and ginsenoside-containing herbal formulations requires further refinement and advancement. (*i*) Mechanistic insights: Although considerable evidence supports the antidepressant-like activity of ginsenosides, the precise molecular and cellular mechanisms remain incompletely to be fully elucidated. Even in studies demonstrating modulation of specific signaling pathways or molecular targets, causal relationships are often not firmly established. Future investigations integrating pharmacological interventions, genetic model systems, and multi omics approaches are needed to clarify the mechanistic basis and therapeutic relevance of ginsenoside-mediated effects. (*ii*) Biotransformation and active metabolites: Growing evidence indicates that the biological activities of many ginsenosides are closely linked to their biotransformation by the intestinal microbiota. Accordingly, further studies should prioritize the identification and structural characterization of individual biotransformation products, determination of their relative bioactivities, and clarification of their specific contributions to the overall antidepressant-like effects of parent compounds. (*iii*) Clinical evidence. To date, clinical studies evaluating the effects of ginseng or isolated ginsenosides on depressive symptoms remain limited. A randomized, double-blind, placebo-controlled trial conducted in which 178 patients with depression received oral YOXINTINE, a formulation containing > 98% PPD, at doses of 200 mg or 400 mg, or placebo, administered twice daily for eight weeks [153]. The study reported significant improvement in depressive symptoms compared with placebo and an acceptable short-term safety profile. While these findings provide encouraging early clinical evidence, the overall body of clinical data on ginsenosides remains preliminary. Therefore, results derived from preclinical and experimental studies require validation in well-designed, large-scale, randomized, placebo-controlled trials to establish their clinical efficacy, safety, and translational potential in the treatment of depression.

Given the high recurrence rate and long‑term morbidity of depression, there remains an urgent need for safe, effective, and sustainable treatment options. We believe that ginsenosides and ginsenoside‑containing herbal formulations represent promising natural sources for the development of stable, efficacious, and low‑toxicity antidepressant agents. Their ultimate potential, however, will depend on systematic animal studies and rigorous clinical investigations designed to bridge preclinical findings with clinical application.

## Data Availability

No datasets were generated or analysed during the current study.

## Abbreviations

4-HNE: 4-Hydroxynonenal
5-HIAA: 5-Hydroxyindoleacetic acid
5-HT: Serotonin
8-OHdG: 8-Hydroxy-2’-deoxyguanosine
ACTH: Adrenocorticotropic hormone
AKT: Protein kinase B
AMPAR: α-Amino-3-hydroxy-5-methyl-4-isoxazole propionic acid receptor
ASC: Apoptosis-associated speck-like protein
ATP: Adenosine triphosphate
Bax: BCL2-associated X protein
CAT: Catalase
CCI: Chronic constriction injury
CMC-Na: Carboxymethyl cellulose sodium
CMS: Chronic mild stress
CNS: Central nervous system
CORT: Corticosterone
COVID-19: Coronavirus disease 2019
COX-2: Cyclooxygenase-2
CREB: Element–binding protein
CRH: Corticotropin-releasing hormone
CRS: Chronic restraint stress
CS: Conditioned stimulus
CSDS: Chronic social defeat stress
CUMS: Chronic unpredictable mild stress
CUS: Chronic unpredictable stress
DA: Dopamine
DCX: Doublecortin
EC: Escherichia coli K1
EPMT: Elevated plus maze test
ERK: Extracellular signal-regulated kinase
fRG: Bifidobacteria-fermented red ginseng
FST: Forced swimming test
GABA: Gamma-aminobutyric acid
GCL: Granular cell layer
GFAP: Glial fibrillary acidic protein
GPx: Glutathione peroxidase
GR: Glucocorticoid receptor
HMGB1: High-mobility group box 1
HO-1: Heme oxygenase 1
HPA: Hypothalamic–pituitary–adrenal
*i.g.*: Intragastric
IL‑1: Interleukin‑1
*i.p.*: Intraperitoneal
IFN-γ: Interferon gamma
iNOS: Inducible nitric oxide synthase
IS: Immobilization stress
IκBα: Inhibitor of κBα
KAT: Kynurenine aminotransferase
KMO: Kynurenine-3-monooxygenase
Kyn: Kynurenine
L-AAA: L-α-aminoadipic acid
LPS: Lipopolysaccharide
MAO: Monoamine oxidase
MAPK: Ras–mitogen-activated protein kinase
MDA: Malondialdehyde
MDD: Major depressive disorder
miR-134: MicroRNA-134
miRNAs: MicroRNAs
MPO: Myeloperoxidase
MyD88: Myeloid differentiation primary response 88
NE: Norepinephrine
NMDAR: N-methyl-D-aspartate Receptor
NSFT: Novelty-suppressed feeding test
OFT: Open field test
*p.o.*: Per os (oral administration)
PI3K: Phosphatidylinositol 3-kinase
PLC-γ: Phospholipase C-γ
PPD: Panaxadiol-type
PPT: Panaxatriol-type
PSD: Postsynaptic density
Quin: Quinolinic acid
ROS: Reactive oxygen species
SCFA: Short chain fatty acid
SD rats: Sprague–Dawley rats
SIRT1: Sirtuin-1
SIT: Social interaction test
SOD: Superoxide dismutase
SPS: Single-prolonged stress
SPT: Sucrose preference test
SYN1: Synapsin I
TDO: Tryptophan 2:3-dioxygenase
TH: Tyrosine hydroxylase
TLR4: Toll-like receptor 4
TNFR: Tumor necrosis factor receptor
TrkB: Tropomyosin receptor kinase B
Trp: Tryptophan
TST: Tail suspension test
UMS: Unpredictable mild stress
US-CS: Unconditioned stimulus
VEGF: Vascular endothelial growth factor
WHO: World Health Organization
